# Antimicrobial Resistance Among Nosocomial Isolates in a Teaching Hospital in Goa

**DOI:** 10.4103/0970-0218.40875

**Published:** 2008-04

**Authors:** US Kamat, AMA Ferreira, R Savio, DD Motghare

**Affiliations:** Department of Preventive and Social Medicine, Goa Medical College Hospital, Bambolim, Goa, India; 2 Department of Microbiology, Goa Medical College Hospital, Bambolim, Goa, India

**Keywords:** Antibiotic usage, antimicrobial resistance, nosocomial infection

## Abstract

**Background:**

Emergence of polyantimicrobial resistant strains of hospital pathogens has presented a challenge in the provision of good quality in-patient care. Inappropriate use of antibiotics in the hospital is largely responsible for this catastrophe. Bacteriological surveillance of the cases of nosocomial infections is crucial for framing an evidence-based antimicrobial policy for a hospital.

**Materials and Methods:**

A prospective study was undertaken among 498 patients from medicine and surgery wards in a tertiary teaching hospital in Goa. The patients were followed up clinico-bacteriologically for the occurrence of nosocomial infections (NI). Antibiotic susceptibility testing was done using Kirby-Bauer disc diffusion method.

**Results:**

The overall infection rate was 33.93 ± 4.16 infections per 100 patients. Urinary tract infection was the most common NI (26.63%), followed by surgical site infection (23.67%), wound infection (23%) and nosocomial pneumonia (18.34%). Ninety-seven percent of the isolates were bacterial, while the others were fungal. More than 80% of the NIs were caused by Gram-negative bacteria, predominantly *Pseudomonas aeruginosa, Escherichia coli* and *Aceinetobacter baumanii*. Almost 70% of the isolates were resistant to all the antibiotics for which susceptibility was tested; the rest were sensitive to amikacin, cefoperazone-sulbactam and other antibiotics including methicillin, co-trimoxazole, teicoplenin, vancomycin and rifampicin, either singly or in combination. The proportion of MRSA was 71.4%. Resistance to a particular antibiotic was found to be directly proportional to the antibiotic usage in the study setting.

**Conclusion:**

Surveillance of nosocomial infections with emphasis on the microbiologic surveillance and frequent antimicrobial audit are critical towards curbing the evil of polyantimicrobial resistant nosocomial infections in a hospital.

Infections acquired in the hospital account for major causes of death, morbidity, functional disability, emotional suffering and economic burden among the hospitalized patients.(1) These nosocomial infections (NI) occur among 7-12% of the hospitalized patients globally with more than 1.4 million people suffering from the infectious complications acquired in the hospital.(2) The issue is further complicated by the emergence of polyantimicrobial resistant strains of hospital pathogens. The microbes have developed the ability to elude the best antimicrobial agents and to counter-attack with new survival strategies that has made the spread of NI easier, and the control even more difficult. Evidence-based antimicrobial prescription policy could help curb the problem; however, surveillance of nosocomial infections is an essential pre-requisite. Differences in the hospital settings preclude the generlistion of results from a hospital to the other hospitals.(3,4)

A prospective study was, therefore, undertaken in a medical college hospital in Goa to estimate the incidence of Nosocomial infections in the medical and surgery wards, and also to study the antimicrobial susceptibility of the hospital isolates.

## Materials and Methods

A prospective study among 498 in-patients, with the hospital stay of more than 48 h in the selected medical and surgical wards of the apex medical teaching institution in Goa, was undertaken during June-December 2005. The patients were followed-up clinico-bacteriologically until they were discharged, or until death during hospitalization or the development of NI. The specific nosocomial infections were diagnosed as per the criteria laid by the Centre for Disease Prevention and Control, Atlanta.(5) Antibiotic susceptibility was tested by the Kirby-Bauer disc-diffusion method. For those with positive culture reports, repeat culture was made weekly, till discharge for an evidence of new infection. Those with the similar isolates with the same antibiogram at subsequent cultures were reported to have a single episode of infection. Isolation of more than two organisms from a sample was considered as an evidence of contamination, and the repeat sample was collected. The antimicrobial sensitivity was tested to the following antibiotics as per the relevance: amoxycillin, augmentin (amoxycillin with clavulinic acid), methicillin, tetracycline, co-trimoxazole, roxithromycin, azithromycin, oxacillin, chloremphenicol, amikacin, gentamicin, tobramycin, netromycin, carbenicillin, teicoplenin, cefadroxyl, cefuroxime, cefoperazone, magnex (cefoperazone with sulbactam), ceftriaxone, cefotaxime, ceftizoxime, ceftazidime, nalidixic acid, norfloxacin, ciprofloxacin, furazolidone, rifampicin, vancomycin, and levofloxacin.

Incidence of NI was expressed as infection percentage(6) (number of patients infected per 100 patients), infection rate(6) (number of episodes of NI per 100 patients) and incidence-density.(7)

## Observations and Discussion

Of the 498 patients, 103 developed 169 episodes of NI. Thus the overall infection percentage was 20.68 ± 3.56%, and infection rate of 33.93 ± 4.16 infections per 100 patients. The overall incidence-density was estimated to be 40.66 ± 7.85 infections per 1000 patient-days. Urinary tract infection was the most common NI (26.63%), followed by surgical site infection (23.67%), wound infection (23%) and nosocomial pneumonia (18.34%). Nosocomial phlebitis and septicemia, respectively, accounted for 4.73% and 3.55% of the total NI.

Two hundred and seventeen biological samples of blood, urine, sputum, pus, wound swabs, and intravenous catheter tips were sent for microbiological assessment during the study period, out of which 164 revealed positive culture reports; the rest five cases showing clinical evidence of NI. In all, 232 isolates were cultured from 164 microbiologically positive cases of NI. Of these, six (2.6%) were fungal while the remaining 226 (97.4%) were bacterial isolates. Table 1 details the five common isolates from the different sites of NI.

**Table 1:** Five common isolates from the different sites of nosocomial infections

Site of Nl	Urinary infection	Pneumonia	Surgical site infection	Skin/soft tissue infection	Septicemia	Phlebitis
Isolates	*E. coli* (49.1%)	*Pseudo. aeruginosa* (47.0%)	*Pseudo. aeruginosa* (22.9%)	*Pseudo. aeruginosa* (29.4%)	*Pseudo. aeruginosa* (57.1%)	*Citro. diversus* (57.1%)
	*Pseudo. aeruginosa* (12.7%)	*Aceineto. baumanii* (17. 7%)	Steph. *aureus* (19.7%)	Steph. *aureus* (23.5%)	*Citro.* diversus (28.6%)	Others† (14.3%)
	Klebsiella (12.7%)	Steph. *aureus* (14.7%)	*Aceineto. baumanii* (14.7%)	*Aceineto. baumani* (16.2%)	*Aceineto. baumani* (14.3%)	-
	Candida (10.9%)	Klebsiella (8.8%)	Klebsiella (13.1%)	*E. coli*(11.8%)	-	-
	*Aceineto. baumanii*(5.5%)	Others* (2.9%)	*E. coli* (11.5%)	*Aceineto. colcoaceticus*(8.8%)	-	-

† Others include one isolate each of *P. aemginosa, S. pyogenes* and C. *frenudii*

* Others include one isolate each of *E. coli, C. freundii*, Proteus and Group D streptococci

More than 80% of the NIs were caused by the Gram-negative Bacteria (GNB). *Pseudomonas aeruginosa, Escherichia coli, Aceinetobacter baumanii* and *Staphylococcus aureus* together constituted more than 70% of the isolates. Increasing importance of GNB in NI has been commented on by a number of investigators.(8,9) *Escherichia coli* was the most common isolate from the cases of urinary tract infection. While *Pseudomonas aeruginosa* dominated the bacteriology of nosocomial pneumonia, surgical site infection, skin and soft tissue infection and septicemia; *Citrobacter diversus* was most commonly implicated in the causation of nosocomial phlebitis. This observation is consistent with the findings of other researchers.(9) Table 2 depicts the nosocomial isolates (fungal isolates excluded) and their antimicrobial susceptibility pattern.

**Table 2:** Nosocomial isolates and their antibiotic susceptibility*

Organisms	Total	Resistant to all†		Sensitive to Amikacin		Sensitive to Magnex‡		Sensitive to Others§	
					
		No.	%	No.	%	No.	%	No.	%
*Pseudomonas*	62	48	77.42	6	9.68	11	17.74	1	1.61
*E. coli*	43	35	81.39	7	16.28	5	11.63	0	0.00
*Staphylococcus aureus*	35	18	51.43	3	8.57	1	2.86	17	48.57
*Aceinetobacter baumanii*	30	22	73.33	0	0.00	8	26.77	1	33.33
*Klebsiella*	21	14	66.67	7	33.33	5	23.81	0	0.00
*Citrobacter diversus*	14	12	85.71	2	14.28	2	14.30	0	0.00
*Citrobacter freundii*	8	2	25.00	1	12.50	6	75.00	0	0.00
*Aceinetobacter colcoaceticus*	8	4	50.00	0	0.00	4	50.00	0	0.00
*Proteus mirabilis*	4	3	75.00	1	25.00	1	25.00	0	0.00
Group D *Streptococci*	1	0	0.00	0	0.00	0	0.00	1	100.00
Total	226	158	69.91	27	11.95	43	19.03	20	8.84

* The table refers to only 226 bacterial isolates

† All the antibiotics for which susceptibility was tested,

‡ Magnex-cefoperazone sulbactam combination

§ Others include Methicillin, Rifampicin, co-trimoxazole, Teicoplenin, Vancomycin

One hundred and fifty-eight (69.9%) isolates were resistant to all the antibiotics for which susceptibility was tested, 11.9% were sensitive to amikacin, 19% were sensitive to cefoperazone-sulbactam, and 8.84% sensitive to other antibiotics including methicillin, co-trimoxazole, teicoplenin, vancomycin and rifampicin. The categories sensitive to amikacin and sensitive to cefoperazone-sulbactam are not mutually exclusive ones, as 7.5% (17 of 226) of the isolates were sensitive to both amikacin and cefoperazone-sulbactam. Maximum sensitivity was thus demonstrated to cefoperazone-sulbactam, followed by amikacin. Increased sensitivity of hospital pathogens to cefoperazone-sulbactam and amikacin, admist widespread antimicrobial resistance has been reported in few studies.(3,8,10)

Among the *Staphylococcus aureus* 28.6% (10/35) were sensitive to methicillin, implying the proportion of methicillin-resistant *Staphylococcus aureus* (MRSA) to be 71.4%. Other studies in India have quoted the prevalence of MRSA ranging from 54.8%(11) to 80.89%.(12) Among the sensitive isolates of *Staphylococcus aureus* 88.2% were sensitive to vancomycin, and all were sensitive to teicoplenin. Emergence of glycopeptide-resistance among *Staphylococcus aureus* has been described by several researchers.(9)

**Figure 1:**
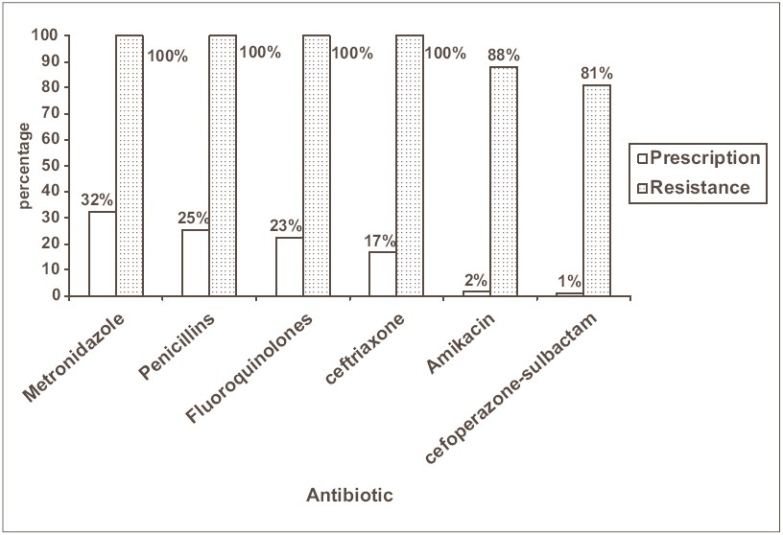
Comparison of antibiotic resistance with the frequency of antibiotic prescription* *The percentages are rounded-off to the nearest whole number

Inappropriate use of antibiotics and consequent selective antibiotic pressure has been incriminated in the genesis of the antibiotic resistant strains in the literature.(9,13,14) Figure 1 depicts the correlation between the specific antibiotic usage in the study wards and the proportion of the isolates resistant to the antibiotic. Antibiotics were prescribed among 72% of the total study subjects; however no attempt was made to probe in to the rationality and appropriateness of the antibiotic prescription.

It is observed that the antibiotics with maximum sensitivity were the ones that were rarely prescribed. An observation similar to this was made with respect to the antimicrobial resistance among *E. coli* in a tertiary hospital in New Delhi, India.(15) The observation reinforces the fact that selective antibiotic pressure escalates the drug resistance and forms a sound basis for the recommending the ‘cycling of antibiotics’.(16) This technique alternates the formulary of antimicrobials between drug classes every couple of months and theoretically reduces the selective pressures of one antimicrobial class.

## Conclusion

High incidence of NIs and the aetiological role played by the polyantimicrobial resistant strains of micro-organisms calls for the revival of the activities of the Infection Control Committee in the hospital. Meticulous surveillance of NIs including the surveillance of hospital isolates and their antibiotic sensitivity patterns could help in formulation of an evidence-based antibiotic policy. It has been stated that antibiotic prescriptions in teaching hospitals, worldwide, are inappropriate in 41-91% of instances.(16) Frequent antimicrobial audit and qualitative research could give an insight in to the current antibiotic prescription practices and the factors governing the same. Regular dissemination of the surveillance information to the health care professionals, feedback from them, and timely corrective actions shall forge a final link in the surveillance cycle.
